# Health Insurance and Differences in Infant Mortality Rates in the US

**DOI:** 10.1001/jamanetworkopen.2023.37690

**Published:** 2023-10-13

**Authors:** Desalyn L. Johnson, Waldemar A. Carlo, A. K. M. Fazlur Rahman, Rachel Tindal, Sarah G. Trulove, Mykaela J. Watt, Colm P. Travers

**Affiliations:** 1 University of Alabama School of Medicine, University of Alabama at Birmingham, Birmingham; 2 Department of Pediatrics, University of Alabama at Birmingham, Birmingham; 3 Department of Biostatistics, University of Alabama at Birmingham, Birmingham; 4 Brooke Army Medical Center, Houston, Texas

## Abstract

**Importance:**

Health insurance status is associated with differences in access to health care and health outcomes. Therefore, maternal health insurance type may be associated with differences in infant outcomes in the US.

**Objective:**

To determine whether, among infants born in the US, maternal private insurance compared with public Medicaid insurance is associated with a lower infant mortality rate (IMR).

**Design, Setting, and Participants:**

This cohort study used data from the Centers for Disease Control and Prevention (CDC) Wide-Ranging Online Data for Epidemiologic Research expanded linked birth and infant death records database from 2017 to 2020. Hospital-born infants from 20 to 42 weeks of gestational age were included if the mother had either private or Medicaid insurance. Infants with congenital anomalies, those without a recorded method of payment, and those without either private insurance or Medicaid were excluded. Data analysis was performed from June 2022 to August 2023.

**Exposures:**

Private vs Medicaid insurance.

**Main Outcomes and Measures:**

The primary outcome was the IMR. Negative-binomial regression adjusted for race, sex, multiple birth, any maternal pregnancy risk factors (as defined by the CDC), education level, and tobacco use was used to determine the difference in IMR between private and Medicaid insurance. The χ^2^ or Fisher exact test was used to compare differences in categorical variables between groups.

**Results:**

Of the 13 562 625 infants included (6 631 735 girls [48.9%]), 7 327 339 mothers (54.0%) had private insurance and 6 235 286 (46.0%) were insured by Medicaid. Infants born to mothers with private insurance had a lower IMR compared with infants born to those with Medicaid (2.75 vs 5.30 deaths per 1000 live births; adjusted relative risk [aRR], 0.81; 95% CI, 0.69-0.95; *P* = .009). Those with private insurance had a significantly lower risk of postneonatal mortality (0.81 vs 2.41 deaths per 1000 births; aRR, 0.57; 95% CI, 0.47-0.68; *P* < .001), low birth weight (aRR, 0.90; 95% CI, 0.85-0.94; *P* < .001), vaginal breech delivery (aRR, 0.80; 95% CI, 0.67-0.96; *P* = .02), and preterm birth (aRR, 0.92; 95% CI, 0.88-0.97; *P* = .002) and a higher probability of first trimester prenatal care (aRR, 1.24; 95% CI, 1.21-1.27; *P* < .001) compared with those with Medicaid.

**Conclusions and Relevance:**

In this cohort study, maternal Medicaid insurance was associated with increased risk of infant mortality at the population level in the US. Novel strategies are needed to improve access to care, quality of care, and outcomes among women and infants enrolled in Medicaid.

## Key Points

**Question:**

Is health insurance type associated with differences in infant mortality rates?

**Findings:**

In this cohort study of more than 13 million infants in the US, maternal private health insurance was associated with a lower risk of infant mortality and adverse infant outcomes compared with Medicaid public health insurance.

**Meaning:**

These findings suggest that there may be opportunities to improve access to care and reduce infant mortality among Medicaid-insured pregnancies in the US.

## Teaser

This cohort study examines whether, compared with public Medicaid insurance, private insurance for the birthing parent is associated with a lower infant mortality rate among infants born in the US.

## Introduction

Infant mortality rates (IMRs) in the US are among the highest in the developed world at least in part because of a combination of racial,^1^ regional,^2^ and socioeconomic disparities.^3^ The IMR is also substantially affected by high rates of preterm birth, which, in turn, may be affected by differences in the reporting of live births at the lowest gestations, and higher gestational age–specific IMRs within the US.^4,5,6^ Medicaid insurance covers pregnancy, perinatal, initial postpartum, and infant care among parents with a gross family income within 138% of the Federal Poverty Level. In 2019, 42.1% of all births in the US were covered by Medicaid insurance.^7^ In some states, pregnant individuals are not automatically made beneficiaries upon conception but must go through an application process. It is possible that this process may result in higher rates of inadequate or delayed prenatal care, which is known to be associated with adverse infant outcomes.^8,9^

In addition, Medicaid Enhanced Prenatal Care Programs that may improve access to prenatal care are associated with lower rates of infant mortality,^10^ raising the question as to whether differences in care related to insurance type are associated with differences in infant mortality owing to underinsurance. Prior studies^11,12,13^ have shown a higher risk of mortality among infants without insurance. Relatively few studies have compared private vs public insurance, focused on infant mortality as a single primary outcome, and adjusted for important confounders. We hypothesized that maternal private health insurance compared with Medicaid public health insurance would be associated with lower rates of infant mortality. In addition, we hypothesized that private health insurance would be associated with lower rates of inadequate prenatal care, and adverse pregnancy outcomes.

## Methods

This cohort study obtained information recorded in the Centers for Disease Control and Prevention Wide-Ranging Online Data for Epidemiologic Research (CDC WONDER) expanded linked birth and infant death records database for 2017 to 2020. The inclusion criteria were all live births from 2017 to 2020 with an obstetrical estimate of 20 to 42 weeks of gestational age.^2^ The study excluded stillbirths, infants with congenital anomalies and those who died as a result of congenital anomalies, those not born within a hospital, those without a recorded method of payment, and those without either private insurance or Medicaid. The Strengthening the Reporting of Observational Studies in Epidemiology (STROBE) reporting guidelines for cohort studies were followed.^14^ This study was reviewed by the University of Alabama at Birmingham institutional review board and was deemed non–human participant research; thus, informed consent was not required, in accordance with 45 CFR §46.

All data were produced by the National Center for Health Statistics and were retrieved from the CDC WONDER expanded database using R statistical software version 4.0 (R Project for Statistical Computing). The primary outcome was the difference in IMR, defined as the number of deaths in the first 365 days after birth per 1000 live births, between private vs Medicaid insurance. Secondary outcome measures included the neonatal mortality rate (defined as the number of the deaths 0 to 27 days after birth per 1000 live births); the postneonatal mortality rate (defined as the number of the deaths from 28 to 364 days after birth per 1000 live births); the timing (trimester in which prenatal care started); the proportion of vaginal breech delivery; the proportion of prenatal steroid exposure; the proportion of low birth weight (<2500 g), extremely low birth weight (<1000 g), preterm (<37 weeks’ gestation), and extremely preterm (<28 weeks’ gestation) births; and the rate of maternal morbidity. Maternal morbidity used the CDC definition that includes maternal transfusion, perineal laceration, ruptured uterus, unplanned hysterectomy, and admission to an intensive care unit. Data on maternal race and ethnicity were obtained from the CDC WONDER database and are included in the analyses as a proxy for systematic racism in the US.

### Statistical Analysis

The statistical analyses compared those with private insurance and those with Medicaid insurance. We used negative-binomial regression to estimate the relative risk (RR) with 95% CI, adjusting for potential confounders, including race, infant sex, multiple birth, any maternal pregnancy risk factors (defined by the CDC as prepregnancy diabetes, gestational diabetes, gestational hypertension, eclampsia, previous preterm birth, and previous cesarean delivery), education level, and tobacco use. To examine differences in the timing of infant mortality by insurance type, a Kaplan-Meier survival analysis was conducted comparing infant mortality (event) by insurance type by day, from birth to 60 days after birth, and by month, from birth to 12 months after birth. A 2-sided *P* < .05 was used to indicate statistical significance. A sample size analysis was not performed for this population-based study. All analyses were performed using SAS statistical software version 9.4 (SAS Institute) between June 2022 and August 2023.

## Results

The study included 13 562 625 infants (6 631 735 girls [48.9%]), of whom 7 327 339 (54.0%) had mothers with private insurance and 6 235 286 (46.0%) had mothers with Medicaid. The private insurance cohort included fewer Black infants, less maternal tobacco use, and fewer female infants than the Medicaid cohort (Table 1). There was a higher rate of multiple births and college degree obtainment in the private health insurance group. There was no difference in the proportion of mothers with at least 1 pregnancy risk factor between the private insurance cohort and the Medicaid cohort (Table 1).

**Table 1.** Baseline Demographics by Maternal Insurance Type

Characteristic	Infants, No. (%) (N = 13 562 625)		*P* value
	Private insurance (n = 7 327 339)	Medicaid (n = 6 235 286)	
Maternal race			
American Indian or Alaska Native	29 954 (0.4)	93 112 (1.5)	<.001^a^
Asian	662 105 (9.0)	248 931 (4.0)	
Black or African American	677 404 (9.2)	1 537 953 (24.7)	
Native Hawaiian or Other Pacific Islander	13 133 (0.2)	28 112 (0.5)	
White	5 776 366 (78.8)	4 132 526 (66.3)	
>1 Race	168 377 (2.3)	194 652 (3.1)	
Sex			
Female	3 578 916 (48.8)	3 052 819 (49.0)	<.001^b^
Male	3 748 423 (51.2)	3 182 467 (51.0)	
≥1 Pregnancy risk factor	4 962 350 (67.7)	4 216 479 (67.6)	.19
Multiple births	271 486 (3.7)	186 882 (3.0)	<.001
Associate’s degree or higher	4 759 063 (64.9)	879 142 (14.1)	<.001
Tobacco user	177 708 (2.4)	686 385 (11.0)	.08

^a^ Value is for Black vs non-Black groups.

^b^ Value is for female vs male birth.

### Infant Mortality

Infants born to mothers with private insurance had a lower IMR compared with infants born to mothers insured by Medicaid (2.75 vs 5.30 deaths per 1000 births; adjusted relative risk [aRR], 0.81; 95% CI, 0.69-0.95; *P* = .009) (Table 2). The neonatal mortality rate did not differ significantly between those who were privately insured compared with those with Medicaid (2.28 vs 2.89 deaths per 1000 births; aRR, 0.96; 95% CI, 0.83-1.11; *P* = .61). The postneonatal mortality rate, however, was lower among infants born to mothers with private health insurance compared with Medicaid insurance (0.81 vs 2.41 deaths per 1000 births; aRR, 0.57; 95% CI, 0.47-0.68; *P* < .001). Kaplan-Meier survival analysis demonstrated that IMR was lower for mothers with private insurance within 60 days after birth and in the first 12 months after birth (Figure).

**Table 2.** Infant Mortality Outcomes by Health Insurance Type

Variable	Infant mortality rate, No. of deaths/1000 births		Adjusted RR (95% CI)^a^	*P* value
	Private insurance	Medicaid		
Overall	2.75	5.3	0.81 (0.69-0.95)	.009
Neonatal	2.28	12.89	0.96 (0.83-1.11)	.61
Postneonatal	0.81	2.41	0.57 (0.47-0.68)	<.001

Abbreviation: RR, relative risk.

^a^ RR for private vs Medicaid insurance was adjusted for race and ethnicity, sex, multiple birth, pregnancy risk factors, educational level, and tobacco exposure.

**Figure.**
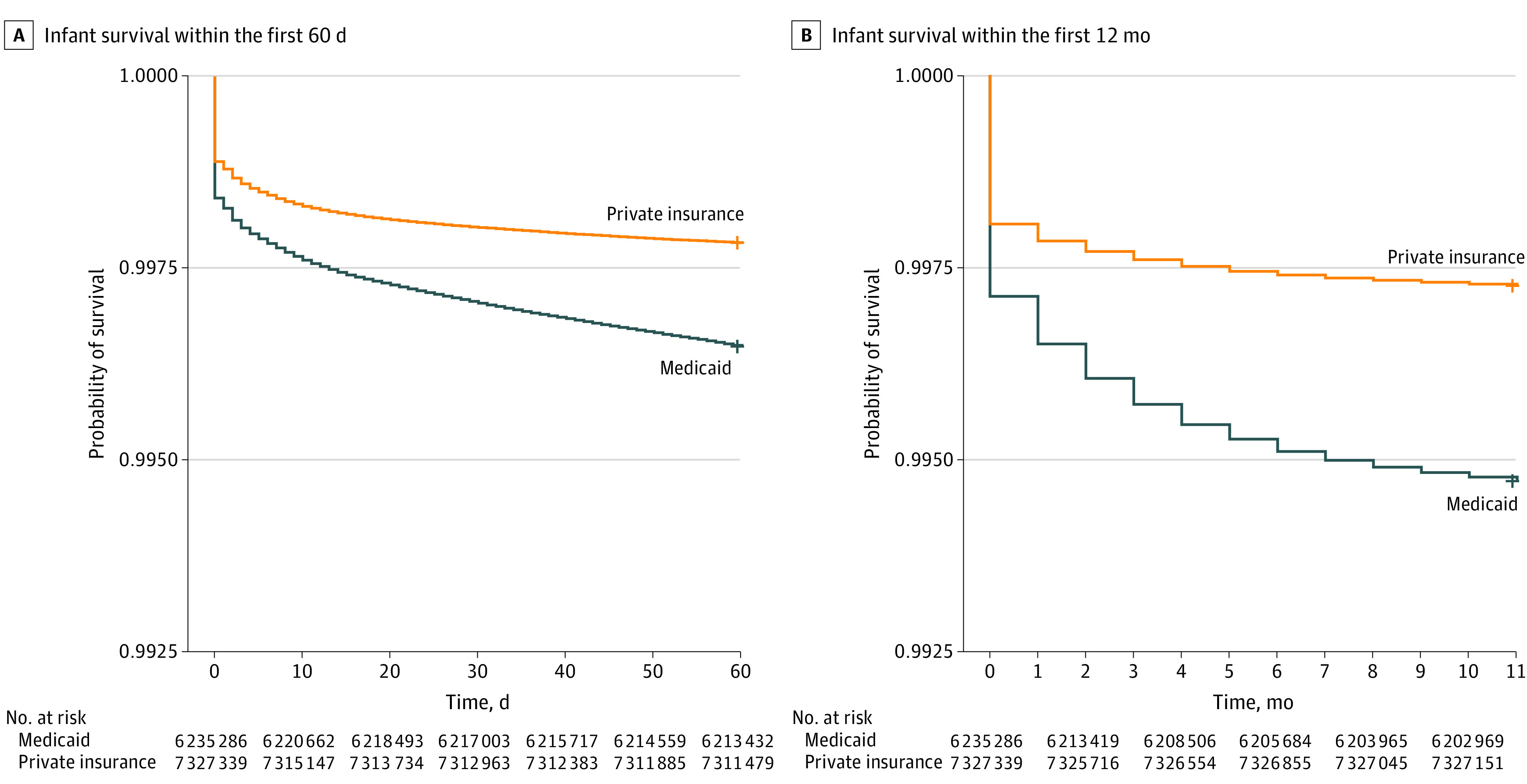
Kaplan-Meier Survival Curves for Infant Mortality by Insurance Type Infants with Medicaid had a lower survival probability than those with private insurance both within the first 60 days after birth (A) and within the first 12 months after birth (B) (both *P* < .001). The cohort includes infants born 2017 to 2020 with an obstetrical estimate of 20 to 42 weeks of gestational age. Stillbirths, infants with congenital anomalies, those not born within a hospital, those without a recorded method of payment, and those without either private insurance or Medicaid are excluded.

### Pregnancy Measures and Outcomes

Mothers of privately insured infants had a higher rate of prenatal care starting in the first trimester compared with mothers of Medicaid insured infants (aRR, 1.24; 95% CI, 1.21-1.27; *P* < .001) (Table 3). Those with private insurance had lower rates of low birth weight birth (aRR, 0.90; 95% CI, 0.85-0.94; *P* < .001), preterm birth (aRR, 0.92; 95% CI, 0.88-0.97; *P* = .002), and breech vaginal birth (aRR, 0.80; 95% CI, 0.67-0.96; *P* = .02). Rates of maternal morbidity (aRR, 0.98; 95% CI, 0.85-1.13; *P* = .75), extremely preterm birth (aRR, 0.91; 95% CI, 0.81-1.04; *P* = .16), extremely low birth weight (aRR, 0.91; 95% CI, 0.79-1.04; *P* = .15), and antenatal corticosteroid use (aRR, 0.97; 95% CI, 0.91-1.03; *P* = .31) did not differ significantly between groups (Table 3).

**Table 3.** Secondary Pregnancy Measures and Outcomes by Insurance Type

Outcome	Infants, No. (%) (N = 13 562 625)		Adjusted RR (95% CI)^a^	*P* value
	Private insurance (n = 7 327 339)	Medicaid (n = 6 235 286)		
First trimester prenatal care	6 279 517 (85.7)	4 160 368 (66.7)	1.24 (1.21-1.27)	<.001
Preterm birth	708 838 (9.7)	743 430 (11.9)	0.92 (0.88-0.97)	.002
Extremely preterm	36 715 (0.5)	45 668 (0.7)	0.91 (0.81-1.04)	.16
Low birth weight	518 967 (7.1)	604 695 (9.7)	0.90 (0.85-0.94)	<.001
Extremely low birth weight	37 402 (0.5)	46 065 (0.7)	0.91 (0.79-1.04)	.15
Vaginal breech delivery	15 370 (0.2)	15 193 (0.2)	0.80 (0.67-0.96)	.02
Maternal morbidity	114 482 (1.6)	71 963 (1.2)	0.98 (0.85-1.13)	.75
Antenatal corticosteroids	252 346 (3.4)	236 418 (3.8)	0.97 (0.91-1.03)	.31

Abbreviation: RR, relative risk.

^a^ RR for private vs Medicaid insurance was adjusted for race, sex, multiple birth, pregnancy risk factors, education level, and tobacco use.

## Discussion

This population-based cohort study found that maternal private insurance was associated with a lower IMR compared with maternal Medicaid insurance. This difference in mortality occurred primarily during the postneonatal time period. In addition, privately insured pregnancies had higher rates of early prenatal care, fewer preterm births, fewer low birth weight births, and fewer vaginal breech deliveries. Our study suggests that health care insurance coverage may be a modifiable risk factor for adverse pregnancy outcomes in the US.

Our findings support smaller or older studies reporting that maternal insurance status was associated with adverse infant outcomes, including IMR, low birth weight birth, prematurity, and complications of prematurity.^11,12,13^ These include an 8-county study^11^ conducted in the San Francisco Bay Area between 1982 to 1986. The findings revealed that a lack of health insurance was associated with increased risk of prolonged hospital stay, transfer to other medical institution, and infant death.^11^ Another study^12^ used data from the Kids’ Inpatient Database for the years 2003, 2006, and 2009. The study included more than 4 million infants (5.4% uninsured) and concluded that uninsured neonates had decreased risk of being admitted in transfer, decreased resource allocation, and increased risk of death in rural settings. A third study^13^ included data for 24 151 infants obtained from the ParadigmHealth database between 2001 and 2005. In that study, infants with Medicaid were associated with lower birth weight, decreased Apgar score at 5 minutes, increased incidence of necrotizing enterocolitis and bacterial sepsis, and increased length of hospital stay. The study also suggested differences in postnatal resource allocation among infants by insurance status, including differences in discharge therapies such as home oxygen and apnea monitors.^13^ In the current study, Medicaid insurance was not associated with differences in antenatal corticosteroid use, a key perinatal care practice among preterm infants.^15^ However, there was a higher rate of breech vaginal birth among Medicaid recipients that may suggest differences in perinatal care practices between groups.^16,17^

In addition to higher rates of infant mortality, higher rates of postneonatal mortality were also seen in the Medicaid cohort in our study. Our findings of higher postneonatal IMRs align with a study^18^ by the National Institute of Child Health and Human Development Neonatal Research Network that found a higher likelihood of post–neonatal intensive care unit discharge deaths among extremely preterm infants without private insurance. The cause of higher postneonatal mortality rates among Medicaid recipients was not evaluated in the current study, but common causes of postneonatal mortality, including sleep-related infant deaths, accidents and injuries, and infection-related deaths, may differ by socioeconomic status.^19^

Although expanded access to public health insurance has been associated with improved adult outcomes,^20^ improving access to prenatal care through public insurance programs may also improve infant outcomes. Medicaid prenatal care expansion among immigrants in Oregon was associated with more prenatal care visits and improved adequacy of prenatal care, a lower extremely low birth weight rate, and a lower IMR, suggesting that increasing access to prenatal care could improve outcomes among Medicaid recipients.^21^ In a previous study^22^ of 9613 women who delivered in North Carolina, Medicaid insurance at delivery was associated with later initiation of prenatal care but no significant difference in rates of preterm birth or low birth weight birth, whereas data on infant mortality were not included. In the current study, we found differences between Medicaid-covered and privately insured women in early initiation of prenatal care. It is not known whether these differences are related to maternal health care–seeking behaviors or to Medicaid application delays.

There are important racial disparities in infant outcomes in the US.^3^ However, genetic ancestry studies^23^ suggest that the basis of race and/or ethnicity as a biological risk factor for adverse health outcomes is limited. In the current study, we adjusted for race as an important confounder related to socioeconomic status and institutional racism in the US.^24^ The assertion that insurance status and related socioeconomic factors may be key factors responsible for differences in perinatal outcomes was supported by a recent study^25^ from the National Institute of Child Health and Human Development Maternal-Fetal Medicine Units Network in which Black full-term neonates had worse perinatal composite outcomes. However, once adjusted for insurance status (private, government funded, or uninsured) the difference was no longer significant.

### Limitations

This study has limitations that should be mentioned. We used a national data set but we did not have access to individual-level data per CDC WONDER data use restrictions. Our data are not generalizable to all subgroups of infants within the US because we excluded infants with congenital anomalies and those born outside of a hospital. Given our large sample size and the relatively few excluded infants, the effect of this bias on the final results is likely modest. Our data only included the source of payment for delivery, which does not consider potential changes to insurance that may have occurred during the pregnancy or after delivery. The resulting bias could underestimate or overestimate the association of infant outcomes with insurance type but should have a small effect. In addition, we did not include self-pay insurance status, which was previously associated with higher rates of infant mortality compared with both mothers with Medicaid and mothers with private insurance using US national data for 2013 and 2017.^26^ In secondary analyses, a lower rate of postneonatal mortality among mothers with private insurance compared with mothers with Medicaid was also reported after adjustment for maternal race, education, age, and marital status.^26^ Using additional data available in the CDC WONDER expanded linked birth and infant death records database, we adjusted for several important antenatal confounders, including maternal race as a proxy for systematic racism, sex, multiple birth, education level, tobacco exposure, and maternal pregnancy risk factors. We did not adjust for postnatal variables because we wanted to limit the adjustments to possible confounders known before birth. However, it is likely that there was residual bias not accounted for in our models, although factors implicated in infant mortality such as gestational age and birth weight may be directly related to insurance type and access to care.^27^

## Conclusions

In this cohort study, maternal private health insurance was associated with a lower IMR compared with Medicaid health insurance. In addition, privately insured pregnancies had higher rates of early prenatal care and fewer preterm and low birth weight births. There are opportunities to improve access to care and pregnancy outcomes among Medicaid insured pregnancies in the US.

## Notes

Supplement.
Data Sharing Statement
