# Supplementary material for: Health Insurance and Differences in Infant Mortality Rates in the US
Source: JAMA Netw Open. 2023 Oct 13;6(10):e2337690. doi: 10.1001/jamanetworkopen.2023.37690 (PMC10576209; doi:10.1001/jamanetworkopen.2023.37690)
Supplement: Supplement. — Data Sharing Statement [file jamanetwopen-e2337690-s001.pdf]

Data Sharing Statement

Johnson. Health Insurance and Differences in Infant Mortality Rates in the US. *JAMA Netw Open*. Published October 13, 2023. doi:10.1001/jamanetworkopen.2023.37690

Data
Data available: No

Additional Information
Explanation for why data not available: All data were produced by the National Center for Health Statistics and retrieved from the CDC WONDER expanded database using R version 4.0.
